# Community- and population-level changes in diatom size structure in a subarctic lake over the last two centuries

**DOI:** 10.7717/peerj.1074

**Published:** 2015-07-02

**Authors:** Elizabeth A. Kerrigan, Andrew J. Irwin, Zoe V. Finkel

**Affiliations:** 1 Environmental Science Program, Mount Allison University, Sackville, New Brunswick, Canada; 2 Department of Mathematics & Computer Science, Mount Allison University, Sackville, New Brunswick, Canada

**Keywords:** Phytoplankton, Climate change, Cell size

## Abstract

Climate change over the last two centuries has been associated with significant shifts in diatom community structure in lakes from the high arctic to temperate latitudes. To test the hypotheses that recent climate warming selects for species of smaller size within communities and a decrease in the average size of species within populations, we quantified the size of individual diatom valves from 10 depths in a sediment core covering the last ∼200 years from a pristine subarctic lake. Over the last ∼200 years, changes in the relative abundance of species of different average size and changes in the average valve size of populations of species contribute equally to the changes in community size structure, but are often opposite in sign, compensating for one another and moderating temporal changes in community size structure. In the surface sediments that correspond to the recent decades when air temperatures have warmed, the mean size of valves in the diatom community has significantly decreased due to an increase in the proportion of smaller-sized planktonic diatom species.

## Introduction

The high latitudes of North America have experienced some of the largest changes in climate over the last century. A range of instrumental, tree ring, and lake and marine sediment records indicate that the arctic has warmed 1–3 °C since the mid-19th century (Overpeck et al., 1997). The subarctic, covering regions from 50–70°N, is amongst the least understood and studied regions in North America (Rouse et al., 1997; Rühland & Smol, 2005). Dendroclimatic reconstruction indicates summer temperatures have risen ∼2 °C since the middle of the 19th century in the subarctic central Yukon and northern and middle Mackenzie Basin (Rouse et al., 1997; Szeicz & MacDonald, 1995). Climate projections indicate that summer temperatures in the subarctic may increase an additional 2–6 °C with a doubling of atmospheric CO_2_ (summary in Rouse et al., 1997).

The subarctic landscape is dotted with numerous lakes and small ponds that are extremely sensitive to climate. Recent warming has been linked with decreases in the duration of river and lake ice cover (Magnuson et al., 2000), alteration in the thermal structure and a lengthening of the growing season in arctic and subarctic lakes (Smol et al., 2005). Model simulations indicate that increases in air temperature will result in an earlier onset and increased intensity and duration of summer stratification (DeStasio et al., 1996; Jones, Sahlberg & Persson, 2010). Changes in the permafrost and hydrological cycle are altering water levels, sediment and nutrient supply and therefore lake transparency (Rouse et al., 1997). Diatoms are often dominant contributors to lake primary production and are extremely sensitive to changes in environmental conditions (Round, Crawford & Mann, 1990). Shifts in diatom and invertebrate community structure from subarctic and arctic lakes have been detected and linked to recent warming (Korhola et al., 2003; Rautio, Sorvari & Korhola, 2000; Smol & Douglas, 2007; Smol et al., 2005). Shifts from benthic to more littoral or periphytic diatoms have been observed in several high arctic lakes, while in deeper arctic and subarctic lakes (>6 m depth) the relative abundance of planktonic *Cyclotella* species often increases at the expense of highly silicified benthic *Fragilaria*, *Achnanthes* or the tychoplanktonic *Aulacoseira* species (Rühland, Paterson & Smol, 2008; Rühland, Priesnitz & Smol, 2003; Smol et al., 2005; Sorvari, Korhola & Thompson, 2002).

A synthesis of over 200 diatom communities from sediment cores of nonacidified, nonenriched lakes across North America shows an increase in the relative abundance of small planktonic *Cyclotella* taxa from less than 10% to between 15 and 45% over the last ∼150 years with concurrent declines in the abundances of heavily silicified *Aulacoseira* taxa and/or small, fragilarioid taxa, depending on the lake and its geographic setting (Rühland, Paterson & Smol, 2008). This shift in diatom community structure, specifically the increase in the relative abundance of *Cyclotella* taxa might signal a decrease in the average size of diatom cells within communities in these Northern Hemisphere lakes, but the average size of the diatom valves and therefore the diatom communities have not been determined. Changes in the size structure of plankton communities could have significant impacts on the trophic transfer efficiency and the structure of higher trophic levels in the food web and alter elemental cycling (Brooks & Dodson, 1965; Daufresne, Lengfellner & Sommer, 2009; Finkel et al., 2010; Hilligsoe et al., 2011; Laws et al., 2000). If there is a shift towards a smaller average size in these diatom communites, this would support a number of recent studies documenting a trend towards smaller mean cell or body size in a number of communities in response to climate warming in both freshwater and marine systems (Daufresne, Lengfellner & Sommer, 2009; Finkel et al., 2007; Hilligsoe et al., 2011; Morán et al., 2009; Winder, Reuter & Schladow, 2009; Yvon-Durocher et al., 2011).

Decreases in the average cell or body size of organisms within communities in response to climate could be caused by taxonomic shifts within communities to smaller-sized species, such as an increase in the proportion of small centric *Cyclotella* species within the community, as well as decreases in the mean size of populations of species, such as a decrease in the mean size of the small *Cyclotella* or other dominant species (Daufresne, Lengfellner & Sommer, 2009). A recent meta-analysis of bacteria, phytoplankton, zooplankton and fish found there were community and population level decreases in cell/body size with recent increases in temperature, leading the authors to conclude that reduced body size is a universal ecological response to warming in aquatic systems (Daufresne, Lengfellner & Sommer, 2009). As a first step to determine if the size structure of Northern Hemisphere diatom assemblages is sensitive to climate warming and if recent climate warming is selecting for both smaller-sized species and decreases in the mean cell size of diatom species we quantify the size of the diatom valves from ten depths covering the last ∼200 years from a representative pristine, subarctic lake that was part of the original Rühland, Paterson & Smol (2008) study.

## Materials and Methods

**Figure 1:**
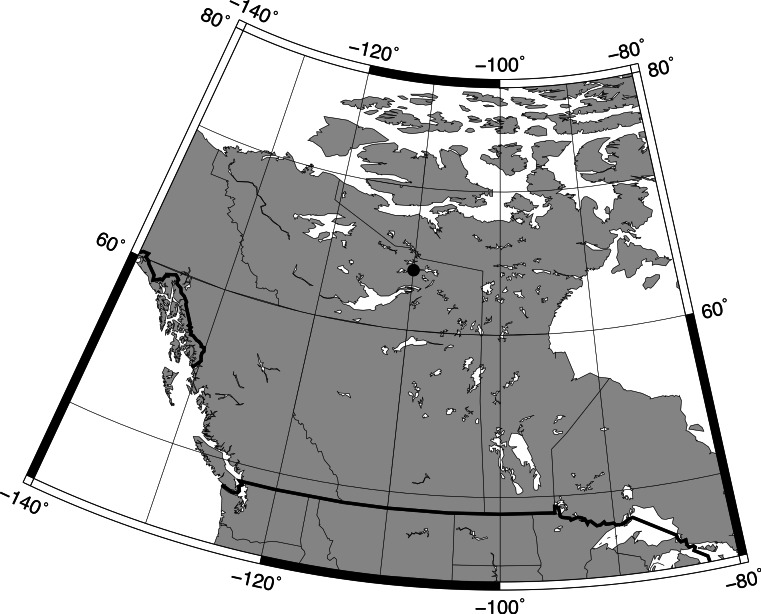
Map showing the location of Slipper Lake in the tundra of the Northwest Territories in Canada.

Slipper Lake (110°W, 64°N; 460 m above sea level) is a remote, tundra lake, located approximately 50 km north of the current tree line in Canada’s Northwest Territories, 300 km from Yellowknife (Fig. 1). At the time of sampling, 1997, the lake had no history of human settlement or disturbance. The lake is 17.0 m deep, has a surface area of 1.9 km^2^ and a pH of 6.4. Climate in this region normally consists of short, cool summers and long, cold winters with a mean annual temperature of −10.5 °C (Rühland & Smol, 2005).

Coring, dating, sampling and diatom preparation were performed using standard procedures as part of the original study by Rühland & Smol (2005). In brief, a 45.5 cm sediment core was retrieved from the deepest part of Slipper Lake (17 m) using a modified KB gravity corer, with an inner diameter of 10.0 cm and a mini-Glew gravity corer with an inner diameter of 4.0 cm. The core was then sectioned on-site, 2 cm for the surface sediments and then at intervals of 0.5 cm for the first 6.5 cm, and then 1.0 cm intervals for the remainder of the core. The top 6.5 cm was dated using ^210^Pb through alpha spectrometry. All the unsupported ^210^Pb was found in the upper 4 to 4.5 cm of sediment. A constant rate of supply model was applied (Appleby & Oldfield, 1978) and dates were extrapolated back to 6.5 cm. The relationship between total ^210^Pb and core depth, the relationship between log ^210^Pb unsupported activity and cumulative dry weight of sediment, and the age-depth profile for the core using the constant rate of supply model are provided in Fig. 2 in Rühland & Smol (2005). Ninety-five percent confidence intervals on estimated ages are larger for older sediments and are typically 10–20 years wide for samples dated 100 years before present, and 8–90 years wide for samples dated 150 years before present (Binford, 1990). Diatoms were cleaned and mounted on glass slides following standard procedures (Battarbee et al., 2001).

A minimum of 300 images of individual diatom valves were taken from slides from each depth using a Canon Powershot A640 camera with a Canon LA-DC58F lens adapter attached to a Ziess Axio Imager A1 light microscope with phase contrast at a magnification of 400x. The size of the diatom valves was determined by manually outlining the area of the diatom valve using a tablet and electronic pen and the program Image-J version 1.44. Image-J was calibrated using a Ziess micrometer. From the outline Image-J provides estimates of the area and perimeter enclosed by the outlined shape and the major and minor axes of the best fitting ellipse. The major and minor axes are used to estimate the diameter and height of centric species, such as *Aulacoseira*, when in girdle view. The measured area of the valve provides a 2-dimensional estimate of the area (*A*) of diatom valves, making no assumptions about the unobserved 3rd dimension. The mean log_10_ area of the ∼300 valves from 10 depths between 0-6.5 cm was calculated to estimate the mean area of all the diatom valves in the community through time. We log-transformed the size measurements before calculating the mean because the observations were approximately log-normally distributed. The mean and 95% confidence interval of area and the other size metrics were computed from the corresponding log_10_ estimates and although the distribution is slightly asymmetric, we report the approximate standard errors for brevity.

For small centric diatoms observed in valve view that are <10 µm in diameter, as well as *Aulacoseira perglabra* var. *perglabra*, *Aulacoseira perglabra* var. *floriniae*, *Aulacoseira lirata* var. *biseriata*, and *Aulacoseira lirata* var. *lirata*, additional images were taken from each depth interval to ensure a minimum of 50 observations for each species/variety at each depth. These additional measurements were made to quantify and determine if the size of these index taxa changed in size over time. It was difficult to definitively identify small centric species to the species level and therefore they were aggregated into a single category (small centrics <10 µm in diameter). Since these species were found in valve view the size of their valves was estimated as the area of the valve. *Aulacoseira* species were predominantly observed in chains in girdle view. All measurements reported are for single valves. Surface area (*SA*) and volume (*V*) of *Aulacoseira* valves were calculated from the major (an estimate of the diameter of the valve when the species is in girdle view) and minor (an estimate of the height of the valve in girdle view) axes assuming a cylindrical shape with the unobserved third dimension equal to the major axis. For the *Aulacoseira* species and varieties, area (*A*) is compared to: the area estimated from the product of the major and minor axes (*A_M_*), the estimate of valve surface area (*SA*), and the volume enclosed by the valve (*V*).

We used an analysis of variance to test for changes in the log_10_ size of the whole diatom community (*A*) as well as the individual populations of small centrics (including the small *Cyclotella* spp.) and *Aulacoseira* species and varieties (*A*, *A_M_*, *SA*, *V*) over depth (time). To determine if the area of these populations changed between the surface sediment compared with all measurements below the surface we used a t-test. We tested for a linear relationship between log_10_ size of the whole diatom community (*A*) and their changes over time with a regional dendrochronological temperature record and its change over time (Szeicz & MacDonald, 1995) using ordinary least squares regression. We computed an average temperature for each layer sampled in the sediment core. All statistical tests were conducted with the statistical package R version 2.15 (R Development Core Team, 2013).

We analyzed the relative contribution of changes in the proportion of the major taxa (species shifts) and changes in the mean size of the major taxa (population cell size shifts) to the change in the average log size of diatom valves in the whole community (log_10_*A*) over time. For this analysis we computed the change in community log area per year (Δlog_10_*A*) between samples at each depth. We partitioned the community log_10_*A* into the sum of the mean log area, *A_i_*, of the three major taxonomic groups that make up the diatom community (small centric species <10 µm in diameter, the *Aulacoseira* species and varieties, and all other species) weighted by their relative abundance in the community, *P_i_*, so that log_10_*A* = Σ*P_i_A_i_*. Then we compute the contribution to the temporal change in community log_10_*A* due to changes in either log area (mean valve size) or relative abundance of each species group using the product rule, Δlog_10_*A* = ΣΔ*P_i_A_i_* + *P_i_*Δ*A_i_* + Δ*P_i_*Δ*A_i_*, and split the last term (Δ*P_i_*Δ*A_i_*) equally between the change due to changes in proportion (Δ*P_i_A_i_* + Δ*P_i_*Δ*A_i_*/2) and the change in log_10_ valve area (*P_i_*Δ*A_i_* + Δ*P_i_*Δ*A_i_*/2) for each species group.

The net change in community log_10_*A* was always less than the sum of the absolute changes due to each of the six components analyzed: (1) the proportion of the community that are small centrics, (2) the proportion of the community that is *Aulacoseira*, (3) the proportion of the community that are not small centrics or *Aulacoseira* (all other species), (4) the average valve size of the small centric valves <10 microns in diameter, (5) the average valve size of the *Aulacoseira* species and varieties, and (6) the average size of the valves that are not small centrics or *Aulacoseira*. The six components contributing to Δlog_10_*A* can differ in sign and magnitude, we therefore summarized the total compensatory effect (*C*) amongst the six factors as: one minus the sum of the absolute value of the change in the six components over time divided by the net change in community log_10_*A* over time. We used a two-sample t-test to compare the fraction of the temporal changes in mean community log_10_*A* attributable to aggregate changes in mean size of the major taxa versus the change in relative proportion of the major taxa.

## Results

### Area, surface area, and volume of the diatom valves

**Figure 2:**
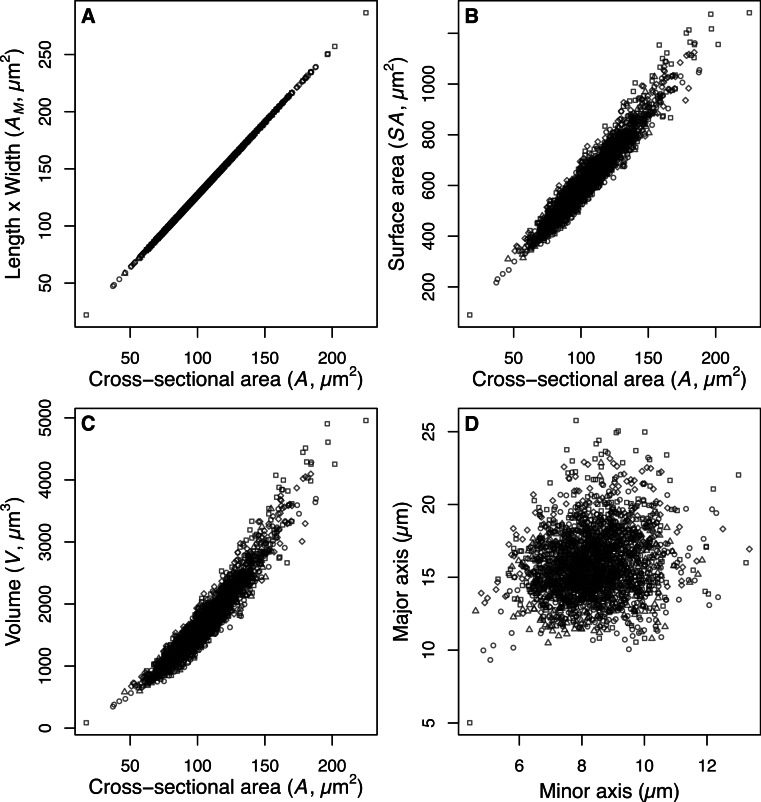
Size and shape of *Aulacoseira* valves. (A) Comparison of measurements of valve area (*A_M_*, µm^2^), (B) surface area (*SA*, µm^2^), and (C) volume (*V*, µm^3^) of *Aulacoseira* valves with the cross-sectional area (*A*, µm^2^) and (D) the major (diameter, *W*) and minor (mantle height, *L*) axes in valve view. Symbols for each species/variety are *A. lirata* var. *biseriata*, circle; *A. lirata* var. *lirata*, square; *A. perglabra* var. *floriniae*, diamond; *A. perglabra* var. *perglabra*, triangle.

The estimate of average size of the diatom community (*A*) is derived from measurements of the two-dimensional area (µm^2^) enclosed by the outline drawn around the images of intact valves. This measurement of valve area makes no assumptions about the unobserved 3rd dimension of the valves. The majority of small centric species were imaged in valve view, and therefore the estimated valve area does not include the mantle depth (the height of the valve surrounding the valve face). In contrast the dominant *Aulacoseira* species examined were imaged predominantly in girdle view and therefore the estimate of area includes the mantle height and the diameter of the valve. Consequently we are able to estimate the 3-dimensional shape for *Aulacoseira*, but only the 2-dimensional shape of the small centric species and the average size of diatoms making up the community assemblage. Our estimates of valve area (*A*) for *Aulacoseira* are positively correlated with our alternate estimate of valve area *A_M_*, our estimate of surface area *SA*, and our estimate of *V* for the dominant *Aulacoseira spp*. (Fig. 2). The area determined from the outline of the valves, *A*, is systematically smaller but linearly correlated with *A_M_*, since the rectangular shape implied by *A_M_* (length × width of the valves) includes extra area not included in the hand tracing of the valve perimeter that was used to estimate *A* (Fig. 2).

### The mean size of the diatom community and relationship with depth in the sediment core and local temperature

**Figure 3:**
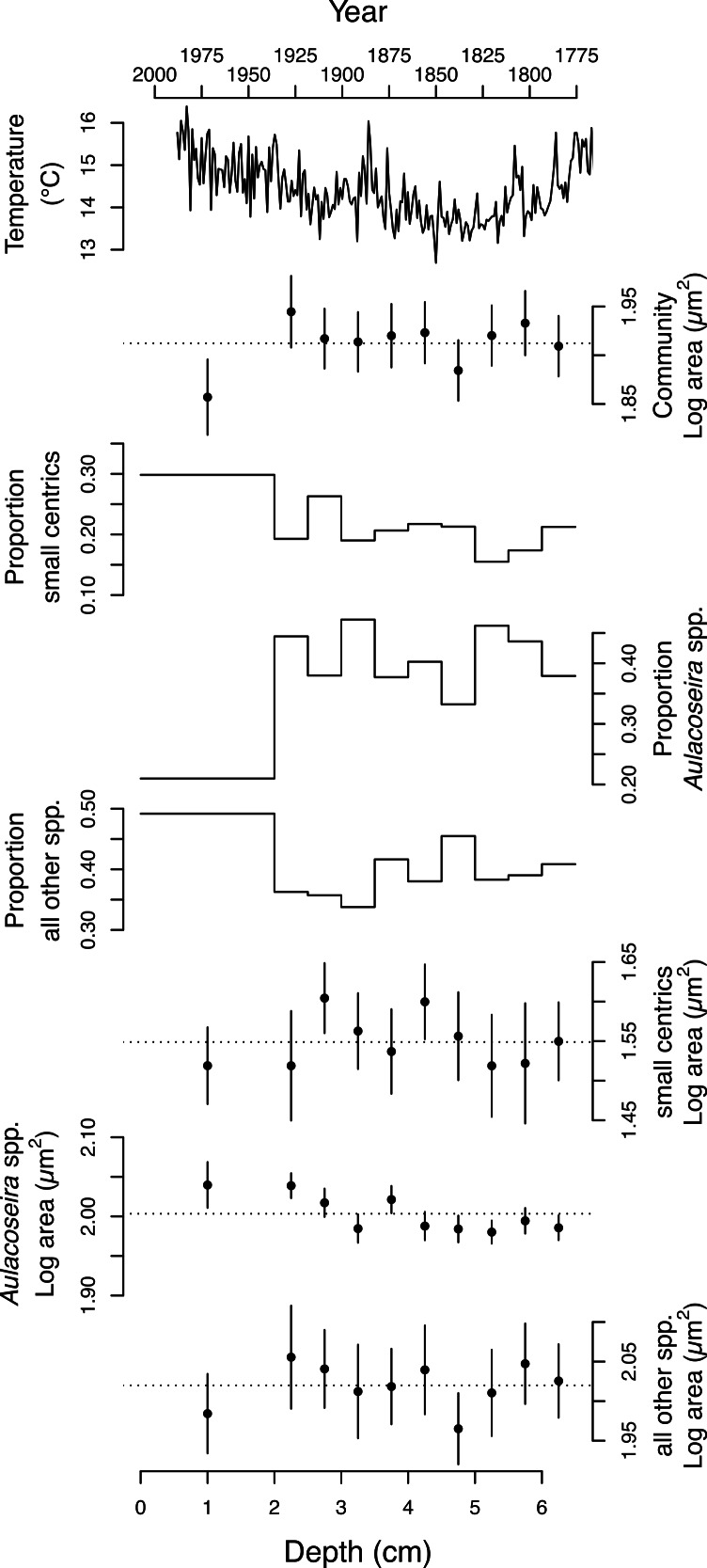
Change in the size and taxonomic structure of the diatom community and selected sub-populations over the last ∼200 years. From top to bottom: regional spring-summer temperature reconstruction (Szeicz & MacDonald, 1995), the average log_10_ area (µm^2^) of valves from the diatom community, the proportion of small centric diatoms with diameters <10 µm in the community; the proportion of *Aulacoseira* spp. in the community, the proportion of all other species in the community, the average log area (µm^2^) of the small centric diatoms with diameters <10 µm, the average log area (µm^2^) of dominant *Aulacoseira* species, the average log area (µm^2^) of all the other species in the community. Errors bars represent 95% confidence intervals.

The 4,899 diatom valves analyzed from Slipper Lake range over ∼2.5 orders of magnitude in area, from a minimum of 7.4 to a maximum of 2169.5 µm^2^. The smallest valves observed at each depth, 7.4–12 µm^2^, are unidentified centric diatoms. The largest valves (546.5–2169.5 µm^2^) are associated with rarely observed *Eunotia* and unidentified naviculoid species. The distribution of valve areas is right-skewed log-normal. The mean area of the valves (*A*), including all depths sampled is 81.7 ± 1.95 (2 se) µm^2^. Over the ten depths sampled the mean area of the valves in the diatom community ranges from 72 to 88 µm^2^. The mean log_10_ area of the diatom valves from the community is statistically indistinguishable across most of the depths analyzed with the exception of the surface sediments (∼last 50 years) when the log_10_ mean area becomes significantly smaller (Table 1 and Fig. 3). There is no significant linear relationship between the mean area of the diatom valves in the community (log *A*) or proportion of small centrics in the community and local spring-summer temperatures over the last two centuries (linear regression, *p* > 0.05).

**Table 1:** Significance tests on the change in the mean size of valves in the diatom community and specified sub-populations over time. The *F*-scores report the results of ANOVAs on log_10_ size to test the null hypothesis that size is independent of depth (time). The *t*-test reports the comparison of the mean size in the surface sediments relative to the nine deeper (older) depths. The mean was calculated on log transformed data since the size data are approximately log-normally distributed. Several estimates of mean size (±2 SE) were analyzed: the cross-sectional valve area (A, µm^2^), *A*, area (length *x* width, *A_M_*, µm^2^), surface area (*SA*, µm^2^), and volume (*V*, µm^3^) of the valves. See the Methods for additional details.

	Average	2 SE	ANOVA	*t*-test
**Average size of the diatom community**				
Log area (A)	81.7	2.0	*F*_9,3069_ = 2.3, *p* = 0.01	*t* = − 3.05, *p* = 0.002
**Centrics <10 µ**m** in diameter**				
Log Area (A)	35.5	1.4	*F*_9,643_ = 1.5, *p* = 0.14 (n.s)	*t* = − 1.43, *p* = 0.16 (n.s.)
***Aulacoseira lirata var. biseriata***				
Log area (A)	98.4	1.7	*F*_9,600_ = 7.82, *p* ≤ 10^−10^	
Log area (*A_M_*)	125.3	2.2	*F*_9,600_ = 7.82, *p* ≤ 10^−10^	
Log V	1,476	40	*F*_9,600_ = 6.66, *p* ≤ 10^−8^	*t* = 4.43, *p* < 10^−4^
Log SA	572	10	*F*_9,600_ = 6.79, *p* ≤ 10^−8^	
***Aulacoseira lirata var. lirata***				
Log Area (A)	105.6	2.0	*F*_9,585_ = 7.49, *p* ≤ 10^−9^	
Log Area (*A_M_*)	134.5	2.5	*F*_9,585_ = 7.49, *p* ≤ 10^−9^	
Log V	1723	50	*F*_9,585_ = 5.89, *p* ≤ 10^−7^	*t* = 3.56, *p* < 0.001
Log SA	634	12	*F*_9,585_ = 5.94, *p* ≤ 10^−7^	
***Aulacoseira perglabra var. floriniae***				
Log Area (A)	109.6	2.1	*F*_9,527_ = 4.57, *p* ≤ 10^−5^	
Log Area (*A_M_*)	139.6	2.6	*F*_9,527_ = 4.57, *p* ≤ 10^−5^	
Log V	1,862	51	*F*_9,527_ = 4.72, *p* ≤ 10^−5^	*t* = 3.98, *p* < 0.0002
Log SA	667	12	*F*_9,527_ = 4.74, *p* ≤ 10^−5^	
***Aulacoseira perglabra var. perglabra***				
Log Area (A)	96.6	1.7	*F*_9,534_ = 3.77, *p* ≤ 10^−3^	
Log Area (*A_M_*)	123.0	2.1	*F*_9,534_ = 3.77, *p* ≤ 10^−3^	
Log V	1,436	38	*F*_9,534_ = 4.11, *p* ≤ 10^−4^	*t* = 1.77, *p* = 0.08 (n.s)
Log SA	562	10	*F*_9,534_ = 4.07, *p* ≤ 10^−4^	

### Changes in mean size and relative percent abundance and of the dominant taxa over time

**Figure 4:**
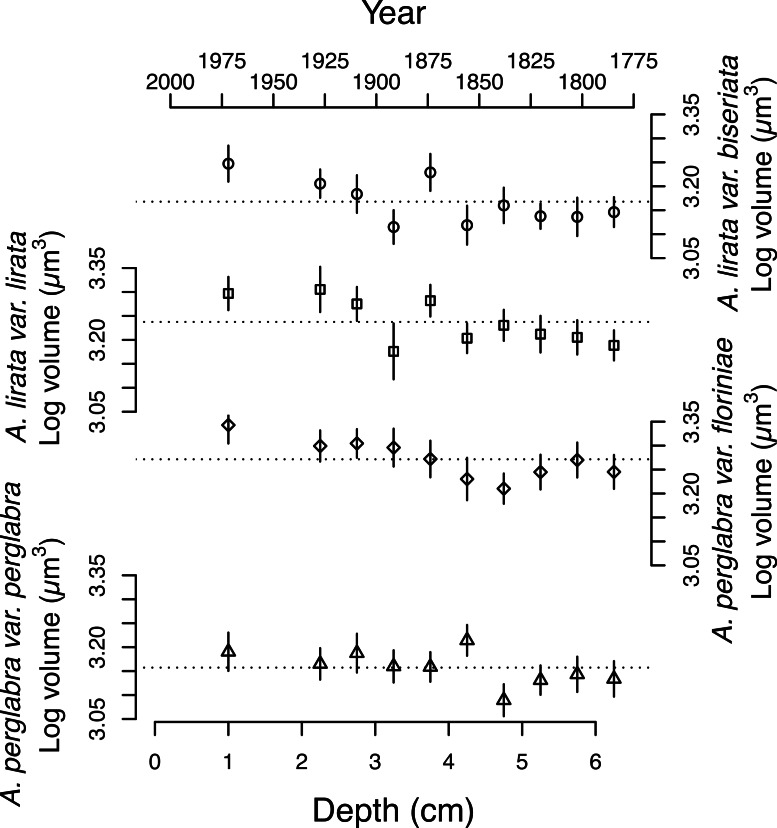
Changes in the average log volume (µ**m^3^**) of valves of the four dominant *Aulacoseira* species over the last ∼200 years. Symbols for the four varieties/species of *Aulacoseira* as in Fig. 1.

The average area of the small centric diatoms <10 µm in diameter in valve view is 35.5 ± 1.4 (2 SE) µm^2^, and does not vary significantly with time/depth (Table 1). The average area of *Aulacoseira* valves range from 96.6 to 109.6 µm^2^, and in volume from 1,436 to 1,723 µm^3^, depending on the species and variety. In contrast with the small centric species, the average valve sizes (*A*, *A_M_*, *SA*, and *V*) of *Aulacoseira lirata* var. *biseriata*, *A. lirata* var. *lirata*, *A*. *perglabra* var. *floriniae* but not *A*. *perglabra* var. *perglabra* does change significantly with depth/time (Table 1); increasing in *A*, *SA*, and *V* since ∼1800 AD (Fig. 4). There are small, but significant, changes in the aspect ratio of *Aulacoseira* with depth (ANOVA, *p* < 0.05), but no trend towards smaller or larger aspect ratios through time across the species and varieties. The aspect ratio of the *Aulacoseira* valves (diameter:mantle height) ranges from 1.82 for *Aulacoseira lirata* var. *biseriata* and *A*. *florinae* var. *perglabra* to 2.0 and 2.1 for *A. lirata* var. *lirata* and *A. perglabra* var. *floriniae*, respectively. The average valve size associated with the other species examined (excluding the small centrics <10 µm and *Aulacoseira* sp.) vary from 92 to 105 µm^2^ across depth but exhibit no consistent trend over depth/time (Fig. 3).

An increase in the relative abundance of small centric diatoms with diameters <10 µm is often associated with a decrease in the relative abundance of *Aulacoseira* species over time (Fig. 3). Small centric species with diameters <10 µm range from 16–21% abundance in the community in the earlier part of the sediment record (3–6.5 cm) to 30% in the surface sediments, indicating there is an overall increase in the relative abundance of small centric species in the community over the last two centuries. In concert, the percent abundance of *Aulacoseira* decreases to 21% in the surface sediments from an average 41 ± 5 % (1 SD) from 2–6.5 cm depth (Fig. 3). The rest of other species (excluding the small centrics and *Aulacoseira* species) account for between 34 to 49% of the relative numerical abundance of the community.

### The contribution of change in taxonomic structure and mean size of the dominant taxa to temporal change in community size structure

The average size of valves in the diatom community log_10_*A* can be decomposed into a weighted sum of the proportion of different taxa of different size and the average size of these taxa. Over all the depths sampled, changes in the proportion of small centric species <10 µm in diameter was the most important or second most important factor contributing the temporal change in community log_10_*A* (Table 2). In aggregate, the temporal change in the proportion of taxa of different size and changes in their mean size (small centric species <10 µm in diameter, the *Aulacoseira* species and varieties, and all other species) each account for an average of ∼50% of the change in community log_10_*A* (the absolute value of the change in relative proportion of taxa: 0.48, 95% confidence interval: 0.27–0.69; the absolute value of the effect of the mean size of taxa: 0.52, 95% confidence interval: 0.33–0.70).

**Table 2:** Changes in the mean size of diatom valves in the community (log_10_*A*) over time (with depth D, cm) in the sediment core. For each depth, the average change in log_10_*A* per year is calculated between each depth and the depth below it (Δ*A*). This change is decomposed into six components: the changes in community log_10_*A* due to changes in proportion (*P*) and mean size (*S*) of each of the major taxonomic groups analysed. Note Δ*A*, P and S have been multiplied by 10^4^. The net change in Δ*A* is less than the sum of the absolute magnitudes of the change in the six components, and the extent to which the individual changes cancel out is reported as the compensatory effect (*C* = 1 − the sum of the absolute value of the six component changes divided by the net change in community log_1_0*A*).

D	Δ*A*	C	Small centrics <10 µm in diameter		*Aulacoseira*		Other diatom species	
			P	S	P	S	P	S
0–2	−21.33	19%	−10.94	0.01	−5.41	0.07	2.36	−7.43
2–2.5	15.47	59%	13.98	−10.92	4.02	4.99	0.41	2.99
2.5–3	1.87	95%	−13.48	5.29	−4.51	7.79	1.23	5.56
3–3.5	−3.56	86%	3.40	2.87	4.42	−8.74	−4.20	−1.32
3.5–4	−1.73	93%	2.13	−7.46	−1.16	7.33	2.15	−4.71
4–4.5	21.73	34%	−0.75	5.24	4.00	0.76	−4.94	17.40
4.5–5	−20.10	43%	−12.41	3.86	−4.50	0.87	2.72	−10.60
5–5.5	−7.13	59%	4.33	−0.28	0.79	−3.58	−0.39	−7.99
5.5–6	13.24	40%	8.09	−3.02	2.58	2.01	−1.31	4.88
6–6.5	−21.33	NA	NA	NA	NA	NA	NA	NA

The relative contribution of changes in the proportion and the mean area of the major taxa on changes in community log_10_*A* varies over time. For example, in the surface sediments, 51% of the decrease in community log_10_*A* over time is due to the increase in the proportion of small centric species (51% = 10.94/21.33, see Table 2), 25% is due to the decrease in the proportion of *Aulacoseira* species, and 35% is due to the decrease in the mean valve size of all the other species. The sum of the magnitude of these components, 51 + 25 + 35 = 111, exceeds 100 because of compensatory changes that are acting to moderate changes in community log_10_*A* over time. In particular there is a compensatory increase in the proportion of species other than the small centrics and *Aulacoseira* species (−11.08% = 2.36/21.33) and an increase in the mean size of the small centrics and *Aulacoseira* species (−0.36% = 0.08/21.33). The relative importance of the proportion and average size of the three major taxonomic groups examined (small centric species, *Aulacoseira* species, and all the other species) to changes in community log_10_*A* over time are very similar in the surface sediments and at 4.5–5 cm where we observe both the largest decreases in the community log_10_*A* over time and smallest community *A* (Table 2). The largest increases in community *A* over time occur at 2–2.5, 4–4.5 and 5.5–6 cm in the sediment core. These increases in log_10_*A* are associated with a decrease in the proportion of small centrics and/or the other species not identified, an increase in the proportion and average size of the *Aulacoseira* species and varieties, and/or an increase in the mean size of the valves in the community that are not small centrics or *Aulacoseira* (Table 2).

The net change in community log_10_*A* at each depth exceeds the sum of the absolute changes due to the changes in the proportion of the populations within the community and the average valve size of these populations (Table 2). In other words, changes in the mean size and proportion of any single component can be compensated for by changes in the other components. The total compensatory effect (*C*, one minus the sum of the absolute value of the six component changes divided by the net change in community log area) varies from 19 to 95% of the net temporal change in community log_10_*A*, with an average value of 42 ± 28 (1 SD) % (Table 2).

## Discussion

Slipper Lake, like many lakes across the northern hemisphere, exhibits a significant change in diatom community structure over the last century, including a >10% increase in the relative abundance of the small centric diatoms and ∼20% decrease in the dominant *Aulacoseira* species and varieties over the last half century (Rühland, Paterson & Smol, 2008; Rühland & Smol, 2005; Sorvari, Korhola & Thompson, 2002). These shifts in taxonomic structure are associated with changes in the average size of diatoms (log_10_ mean valve area) within the community (Table 1 and Fig. 3). The largest decreases in community log_10_*A* occur in the surface sediments and in the early 1800s (4–4.5 cm in the sediment core) and are due predominantly to an increase in the relative abundance of the smallest diatom species (<10 µm in diameter) and a decrease in the relative abundance of the larger *Aulacoseira* species and varieties (Tables 1 and 2). The shift towards smaller average diatom cell size at the community level over recent decades is consistent with a large number of studies that document shifts towards smaller average organism size in freshwater and marine communities in response to warming (Daufresne, Lengfellner & Sommer, 2009; Finkel et al., 2007; Hilligsoe et al., 2011; Morán et al., 2009; Winder, Reuter & Schladow, 2009; Yvon-Durocher et al., 2011). We find that recent decrease in community log_10_*A* due to the increase in the relative proportion of small centric species is moderated by the increase in average size of the larger *Aulacoseira* species and varieties. The increase in the proportion of smaller-sized diatom species over recent decades in Slipper Lake may be a general response of diatom communities to warming (Hutchinson, 1957; Rühland, Paterson & Smol, 2008; Winder, Reuter & Schladow, 2009) and is consistent with Bergmann’s rule: smaller-sized species are more prevalent in communities in warmer environments (Bergmann, 1847).

The relationship between regional climate warming and the size structure of the diatom community is not simple. We find no consistent directional trend between reconstructed temperature and the average size of the community or individual populations examined over the last ∼150 years (Figs. 3 and 4). It has been hypothesized that climate warming not only selects for smaller-sized populations of species within communities but also a decrease in the mean size of individual species (Atkinson, Ciotti & Montagnes, 2003; Daufresne, Lengfellner & Sommer, 2009). We find this is not always the case. In Slipper Lake, over all the depths sampled, changes in the relative abundance of taxa of different average size and changes in average size of the dominant taxa are approximately equally responsible for the changes in log_10_ average size of valves in the diatom community (Table 2). At any particular depth the comparative role of changes in the relative abundance of taxa of different size versus the changes in the average size of the dominant taxa varied. In recent decades the major diatom taxa either do not significantly change in average size or exhibit an increase in average size. For example, there is no significant change in the mean size of the small centric diatom species <10 µm, 34.5 ± 3 µm^2^ (1 SD), over the time-series and the mean valve size of three of the four dominant *Aulacoseira* species and varieties increase significantly since ∼1850 AD with warming (Table 1 and Fig. 3). Over the first half of the 20th century (depth 2.5–3 cm in the core) the increase in the average size of the *Aulacoseira* species and varieties compensates, in part, for coincident increases in the proportion of small centric taxa, moderating the change in the average size of the diatoms in the community (Table 2).

What could account for the increase in the proportion of small centric diatoms, such as *Cyclotella pseudostelligera* and an increase in the average size of *Aulacosiera* over the last several decades? One possibility is that *Aulacoseira* may be responding to changing conditions associated with the early spring or fall overturn while the small *Cyclotella* taxa may be responding to conditions associated with an increase in the stratified period or strength of vertical stratification over the growing seasons. Historically, the increase in the relative abundance of the *Cyclotella* complex over the last several decades has been attributed to an increase in the ice-free season and associated change in the growing season and/or changes in thermal stability and length and strength of vertical stratification in lakes (Rühland, Paterson & Smol, 2008; Rühland & Smol, 2005; Sorvari, Korhola & Thompson, 2002) associated with warming over the last century (Fig. 2). Increases in the duration of stratification can result in lower nutrient concentrations in the surface in the late spring and summer. Lower nutrient concentrations favor smaller species such as *Cyclotella pseudostelligera* relative to larger diatom species (Hutchinson, 1957; Smol, Brown & McIntosh, 1984) due to their lower minimum nutrient requirements for growth and higher nutrient uptake affinity (Aksnes & Cao, 2011; Irwin et al., 2006; Munk & Riley, 1952). Warming in conjunction with increased organic loads could also facilitate increased phosphate availability (Wilhelm & Adrian, 2008), that could result in a decrease in dissolved Si:P and decrease in light availability, potentially favoring smaller diatoms, especially in later spring and summer (Finkel et al., 2009; Kilham, Theriot & Fritz, 1996). In addition, there may be an increase in the supply of atmospheric reactive nitrogen since the late nineteenth century (Holtgrieve et al., 2011); but currently the contribution of inorganic nitrogen addition through precipitation appears to be a small proportion of the total nitrogen budget in some arctic lakes (Whalen & Cornwell, 1985) and the atmospheric deposition of nitrogen does not correlate with the significant changes in diatom community taxonomic structure seen in recent decades (Rühland, Paterson & Smol, 2008). Although we hypothesize stratification and nutrient availability may play a key role in regulating the changes in diatom community size structure in Slipper Lake over the last ∼200 years, the size-structure of plankton communities can be shaped by a balance of many size-dependent processes. Loss rates, such as sinking rate and grazing susceptibility, as well as growth rate and the acquisition of resources are all size-dependent processes that can vary with temperature and corresponding changes in light, nutrient concentrations and nutrient supply ratios (Finkel et al., 2010; Finkel et al., 2009; Kilham, Theriot & Fritz, 1996).

Field studies indicate that the size of *Aulacoseira* species appears to be sensitive to nutrient concentration, although the responses appear to be species-specific (Gomez, Riera & Sabater, 1995; Selbie et al., 2011; Turkia & Lepisto, 1999). High relative abundances of several *Aulacoseira* species have been linked to spring mixing (Round, Crawford & Mann, 1990) and some species are known to initiate blooms under ice (Weyhenmeyer, Westöö & Willén, 2008). Climate warming and increases in the duration of the growing season and stratified period may cause both a decrease in nutrient in the surface layer in the late spring and summer, favoring the smaller centric diatom taxa, and an accumulation of nutrient during transient mixing events in the spring and fall overturn (Kilham, Theriot & Fritz, 1996), perhaps favoring larger-sized *Aulacoseira* species. Higher temperatures associated with recent warming may also facilitate higher remineralization rates, contributing to higher nutrient concentrations in the hypolimnion that would be mixed in the surface during the spring and fall overturn (Whalen & Cornwell, 1985). Increases in permafrost melt and increased intensity of spring run-off might also have led to increased sediment loading and higher nutrient supply to subarctic lakes, especially in the early spring (Whalen & Cornwell, 1985). Changes in the length of *Aulacoseira*’s life-cycle with changing environmental and biotic conditions associated warming may also account for the observed changes in average size (Jewson, 1992; Round, Crawford & Mann, 1990). Currently we lack the data required to determine if the increase in the size of the dominant *Aulacoseira* species over time reflects evolutionary or ecological selection for larger forms and if the change in cell size is associated with increases in nutrient concentrations in the spring or fall overturn in Slipper Lake or other factors.

## Conclusions

A recent meta-analysis of bacteria, phytoplankton, zooplanton and fish found there were community, population and individual level decreases in organism size with recent increases in temperature, leading the authors to conclude that reduced body size is a universal ecological response to warming in aquatic systems (Daufresne, Lengfellner & Sommer, 2009). In Slipper Lake we find a decline in the average size of valves and selection for smaller-sized species within the diatom community in over the last several decades coinciding with warming. Coincidently, and in contrast to the trend found by Daufresne, Lengfellner & Sommer (2009), there has been an increase in the average valve size of the numerically dominant *Aulacoseira* species and varieties. Over the last two centuries changes in the relative proportion and mean size of the major diatom taxa have equally contributed to the changes in community size structure over time. Throughout the time-series the sum of the absolute magnitude of changes in the proportion and mean size of the taxa often vastly exceed the net changes in community size structure, indicating that compensatory changes in the proportion and mean size of taxa commonly moderate change in community size structure over time. Further studies are required to assess the potential mechanisms responsible for the increase in *Aulacoseira* cell size and if continued climate warming will result in further increases in the abundance of small centric diatoms at the expense of *Aulacoseira*, causing a decrease in average size of diatoms in Slipper Lake, and perhaps other similar lakes across the northern hemisphere.

## Supplemental Information

10.7717/peerj.1074/supp-1 Supplemental Information 1 Data for figures This workbook contains three worksheets, one for each of Figs. 2–4.
Sheet 1/Figure 2:
Raw data for sizes of the four Aulacoseira spp/varieties): cross-sectional traced area (*A*), minor axis, major axis, volume (*V*), surface area (*SA*), and *A_M_* (length × width area).
Sheet 2/Figure 3:
Depth resolved mean log area for the community, small centrics, Aulacoseira spp., and other species, together with samples sizes and standard errors of the means. Also the proportion of the community (computed from the ratios of the sample sizes) for small centrics, Aulacoseira spp., and all other species.
Sheet 3/Figure 4:
Depth resolved mean log volume and log surface area (not shown) for four species/varieties of Aulacoseira together with sample sizes and the standard error of the means for each quantity.
